# Dose–response effectiveness of focused shockwave therapy on ultrasonographic muscular properties in patients with stroke exhibiting ankle spasticity

**DOI:** 10.1186/s12984-025-01724-7

**Published:** 2025-08-21

**Authors:** Shu-Mei Yang, Hung-Hsi Lin, Yen-Hua Chen, You-Lin Lu, Chueh-Hung Wu, Wen-Shiang Chen, Meng-Ting Lin

**Affiliations:** 1 Department of Physical Medicine and Rehabilitation, National Taiwan University Hospital, College of Medicine, National Taiwan University, Taipei, Taiwan; 2 Department of Physical Medicine and Rehabilitation, National Taiwan University Hospital Hsin-Chu Branch, Hsinchu, Taiwan; 3 Department of Physical Medicine and Rehabilitation, National Taiwan University Hospital, College of Medicine, National Taiwan University, No.7, Zhongshan S. Rd., Zhongzheng Dist, Taipei City, 100 Taiwan

**Keywords:** Extracorporeal shockwave therapy, Stroke, Spasticity, Pennation angle, Fascicle length

## Abstract

**Background:**

Post-stroke spasticity (PSS) in the ankle plantar flexors leads to abnormal gait, increased energy expenditure, and a higher risk of falls. Ultrasonographic measures, such as muscle fascicle length (MFL) and pennation angle (PA), provide insight into muscle changes associated with spasticity. This study aimed to investigate the dose-dependent effects of focused extracorporeal shockwave therapy (ESWT) on ultrasonographic muscle properties and clinical outcomes in patients with PSS of the ankle plantar flexors.

**Methods:**

This post hoc analysis was based on a double-blind, randomized controlled trial investigating different ESWT doses for post-stroke ankle spasticity treatment. A total of 39 patients with PSS of the ankle plantar flexors were randomized into two groups: the double-dose ESWT group received 4,000 focused shockwave pulses per session, while the control ESWT group received 2,000 pulses per session. Both groups received four ESWT sessions over a two-week intervention period, followed by a 24-week follow-up period for outcome assessments. Outcome measures included ultrasonographic assessments of MFL, PA, and strain elastography, as well as clinical evaluations using the Modified Ashworth Scale (MAS), Modified Tardieu Scale (MTS), passive range of motion (PROM), Timed Up and Go (TUG) test, and Barthel Index at baseline, and at 1, 4, 12, and 24 weeks post-treatment.

**Results:**

No significant within-group changes in PA or MFL were observed for either ESWT group over the 24-week period. Generalized estimation equation analysis showed no significant group effects on PA, MFL, or strain elastography. However, when analyzing all participants, a significant time-related improvement in MFL was identified. In the double-dose ESWT group, MFL was significantly correlated with MTS, PROM, and TUG test, while PA was significantly correlated with MAS. Given that this was a post hoc analysis, these results should be interpreted conservatively.

**Conclusions:**

While PA and MFL did not show significant differences between groups, the double-dose ESWT group exhibited improved clinical outcomes linked to MFL. These findings suggest that increased ESWT dosage may enhance muscle architecture and function in stroke rehabilitation.

**Supplementary Information:**

The online version contains supplementary material available at 10.1186/s12984-025-01724-7.

## Introduction

Patients with stroke experience spasticity, a common complication characterized by velocity-dependent increases in muscle tone due to heightened excitability of muscle spindles [1]. This post-stroke spasticity (PSS) often affects the ankle plantar flexors, leading to impaired dorsiflexor muscle strength, abnormal gait patterns, increased energy expenditure during walking, and a heightened risk of fall [2]. Given that these functional impairments are closely related to underlying muscle changes, it is essential to investigate the architectural alterations that occur in spastic muscles to fully understand their contribution to mobility limitations in patients with stroke. Spastic muscles frequently exhibit altered properties that compromise force production and limit joint movement [3]. Ultrasonographic muscle properties, such as muscle fascicle length (MFL) and pennation angle (PA), are critical for understanding changes in muscle architecture in spastic muscles and their impact on functional outcomes in patients with stroke [4, 5]. Ultrasound elastography is a noninvasive method for evaluating tissue elasticity. Strain elastography measures tissue displacement through compression, whereas shear wave elastography uses acoustic radiation to assess elasticity; both are visualized with color-coded elastograms [6, 7]. These methods have been increasingly applied in musculoskeletal evaluations to provide quantitative information on muscle properties in spasticity. Because these ultrasonographic techniques allow detailed evaluation of muscle characteristics, they can also be used to monitor treatment effects.

In this context, extracorporeal shockwave therapy (ESWT) has emerged as a promising treatment for spasticity [8, 9], potentially modifying muscle architecture and reducing muscle stiffness [10]. ESWT involves the application of high-energy mechanical waves to stimulate tissue repair, enhance blood circulation, and disrupt pain signal transmission [11]. The physiological effects of ESWT on spasticity may include alterations in spinal cord excitability, mechanical vibrations, regulation of nitric oxide, and modifications in passive muscle stiffness [12–16]. Despite these proposed mechanisms, the optimal treatment parameters, including the type of ESWT (focused versus radial), intensity, number of sessions, and dosage, remain unclear [17–20]. Focused ESWT, which penetrates deeper into tissues compared with radial ESWT, may offer different therapeutic benefits [17]. The effects of focused ESWT on ultrasonographic muscle properties include elongated MFL and decreased PA in patients with stroke, with treatment efficacy lasting up to 4 weeks [21].

While ESWT has demonstrated improvements in spasticity through clinical measures such as the Modified Ashworth Scale (MAS) and the Timed Up and Go (TUG) test [22–26], its dose-dependent effects on ultrasonographic muscle architecture, particularly MFL and PA, remain underexplored [10, 21]. Clarifying these effects is essential for optimizing ESWT protocols in clinical practice.

Given this knowledge gap in the current literature, we conducted a post hoc analysis of the parent study [26] to investigate the dose-response effects of focused ESWT on ultrasonographic muscle properties, specifically MFL and PA, in patients with stroke exhibiting ankle spasticity. We hypothesized that higher ESWT doses would result in greater improvements in muscle architecture, which may be associated with better clinical outcomes.

## Methods

### Study design

This secondary analysis is based on our previous RCT, which investigated the dose-response effectiveness of ESWT in treating PSS of the ankle plantar flexor muscles [26]. The original trial was carried out between January 2022 and April 2024 at a tertiary referral hospital in Taiwan. Individuals with stroke-related ankle plantar flexor spasticity were assigned at random to either the double-dose ESWT group, receiving 4000 pulses per session, or the control ESWT group, receiving 2000 pulses per session. Focused shockwave therapy was applied to the affected calf muscles, and both groups received four ESWT sessions administered twice weekly over a two-week period. The trial was conducted in accordance with the Consolidated Standards of Reporting Trials (CONSORT) guidelines, details of enrollment, allocation, follow-up, and analysis are shown in Supplementary Fig. 1 [26].

In this secondary analysis, we specifically aimed to investigate the effects of different ESWT doses on ultrasonographic muscle properties (MFL, PA, and strain elastography) and their associations with clinical outcomes, assessed at baseline, and at the 1st, 4th, 12th, and 24th weeks post-treatment. The study adhered to the Declaration of Helsinki and was approved by the hospital’s research ethics committee. All participants provided written informed consent, and the trial was registered on ClinicalTrials.gov (NCT05878223).

### Blinding and randomization

In the original trial, the participants, outcome assessors (conducted by an independent physiotherapist), and research assistants were blinded to treatment allocation [26]. Randomization was performed using computer-generated permuted blocks of four, with assignments concealed in sealed envelopes opened at enrollment. A separate physiotherapist, responsible for administering the ESWT sessions, and the study coordinator, who managed the randomization, were aware of group assignments. Participants were allocated to either the double-dose or control ESWT group for treatment.

### Participants

Participants were consecutively recruited from the outpatient rehabilitation clinics of a tertiary referral medical center. Eligible patients were adults aged 20 years or older with a history of unilateral cerebral stroke who exhibited ankle plantar flexor spasticity exceeding MAS grade 1. Only patients with stable clinical status and vital signs were considered for inclusion. Participants were excluded if they met any of the following conditions: (1) history of recurrent stroke, intracranial tumors, traumatic brain injury, or other neurological disorders involving the brain; (2) presence of additional central nervous system diseases (such as spinal cord injury or Parkinson’s disease) or musculoskeletal abnormalities that could interfere with spasticity evaluations; (3) diagnosis of malignancy, bleeding disorders, active infections, or use of implanted electronic devices like pacemakers; and (4) significant cognitive deficits or communication disorders such as aphasia. Moreover, individuals who had received focused shockwave therapy or botulinum toxin treatment targeting the plantar flexors within the preceding three months were not eligible for inclusion. These inclusion and exclusion criteria were based on the original trial [26]. Sample size estimation was conducted using G*Power software (version 3.1.9.4). The calculation assumed a medium effect size, a statistical power of 80%, and a significance level of 0.05. To accommodate a potential 10% dropout rate, a minimum of 16 participants per group was required to ensure sufficient power.

### Interventions

Eligible participants were randomized into the double-dose ESWT group or the control ESWT group according to the protocol of the original trial [26]. A focused ESWT device was used and administered by a physiotherapist who had a decade of clinical experience. The double-dose ESWT group received 4000 pulses per session (2000 each to the gastrocnemius and soleus muscles), while the control group received 2000 pulses to the gastrocnemius only. Both groups underwent four ESWT sessions, twice weekly for 2 weeks. ESWT settings included a frequency of 4 Hz and an energy flux density of 0.10–0.134 mJ/mm², with muscle targeting guided by B-mode ultrasound. Gel was applied to facilitate effective energy conduction. No local anesthetics were administered. Post-treatment, participants were permitted to take paracetamol but were advised against non-steroidal anti-inflammatory drugs. All patients continued with traditional rehabilitation, including physical modalities and orthoses, muscle stretching and strengthening, range of motion training, as well as exercises targeting trunk stability, postural control, functional activities, and ambulation [19, 27].

### Ultrasonographic evaluation

Ultrasound examinations were conducted by the first author, a physiatrist with more than 5 years of ultrasound experience, using a SONIMAGE HS2 ultrasound system (Konica Minolta, Tokyo, Japan). Patients were positioned prone with fully extended knees and ankles in neutral flexion. Measurements were focused on the same location of the spastic medial gastrocnemius muscle.

### Ultrasonographic muscular outcomes

**Fig. 1:**
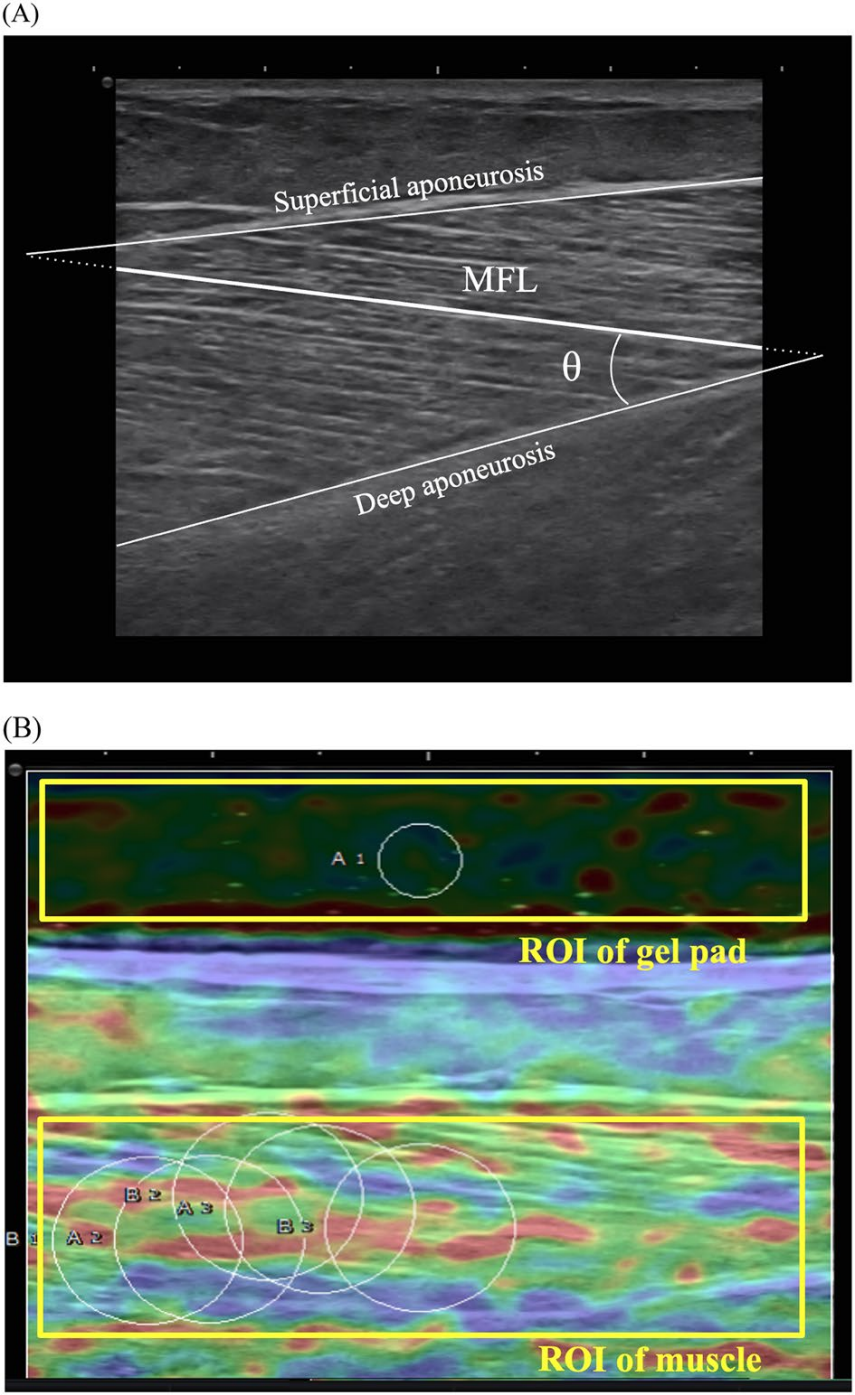
**A** Longitudinal ultrasound images of the medial gastrocnemius muscle for pennation angle (PA) and muscle fascicle length (MFL) measurement. The top solid white line denotes the superficial aponeurosis, while the bottom solid white line indicates the deep aponeurosis. MFL encompassed the fascicle length from deep to superficial aponeuroses. The PA was marked as θ, the angle between the MFL and the deep aponeurosis. **B** The strain elastography image of the medial gastrocnemius muscle. The region of interest (ROI) was designated for the acoustic gel pad and a specific section of the treated gastrocnemius muscle

The outcomes of ultrasonographic muscle properties, including MFL, PA, and strain elastography at the ankle plantar flexor muscles, were measured at baseline and at the 1st, 4th, 12th, and 24th weeks. These measurements were performed using an L18-4 linear probe (SONIMAGE HS2, Konica Minolta, Tokyo, Japan) ultrasound device. B-mode ultrasound images provided a longitudinal view of the scanned muscle. MFL and PA were measured at the midpoint of each muscle belly, with MFL measured between the superficial and deep aponeuroses and PA calculated as the angle between the muscle fascicle and the deep aponeurosis (Fig. 1A) [28]. Three measurements were taken for each parameter, and the average value was used.

Strain elastography used B-mode and elastography modes with the L18-4 linear probe. The scanning methodology is detailed in our previous study [26]. Briefly, the medial gastrocnemius muscle on the spastic side was first scanned transversely using B-mode ultrasound. Subsequently, strain elastography was conducted by applying rhythmic, light compressions with the ultrasound probe to produce tissue deformation. Real-time elastographic images were collected, ensuring a stable strain graph and consistent color distribution during the compression-relaxation cycles. The strain ratio (SR) was calculated as the ratio of strain in the reference material (Aquaflex gel pad, Parker Laboratories, USA) to that in the target muscle, where a higher SR indicates a stiffer muscle and a lower SR indicates a softer muscle. The regions of interest were standardized at 18 × 30 mm for the muscle and 4 × 30 mm for the reference object. Each SR measurement was repeated three times, and the average value was used for analysis (Fig. 1B).

Prior to the trial, intra- and inter-rater reliability were assessed using the intraclass correlation coefficient (ICC). For intra-rater reliability, a two-way mixed-effects model with absolute agreement was used to evaluate the consistency of repeated measurements by the same rater. For inter-rater reliability, a two-way random-effects model with absolute agreement was applied, considering raters and subjects as random samples. The reliability analysis demonstrated excellent measurement consistency both within and between raters. The intra-rater reliability demonstrated an ICC of 0.840, indicating excellent repeatability. The inter-rater reliability yielded an ICC of 0.904, confirming a high degree of agreement between raters.

### Clinical outcomes

The clinical assessments were performed at baseline and subsequently at weeks 1, 4, 12, and 24. These assessments included the MAS, Modified Tardieu Scale (MTS) angles, passive range of motion (PROM) of the ankle plantar flexor muscles, the TUG test, and the Barthel Index. Detailed positions and descriptions of each clinical outcome are available in the methodology section of our previous RCT [26]. Briefly, participants were assessed in a prone position with their knees fully extended and ankles positioned neutrally. They were then instructed to move their ankles through their full range of motion, from maximum plantarflexion to maximum dorsiflexion. Spasticity was assessed using the MAS, which grades resistance to passive stretch on a scale from 0 (no increase in muscle tone) to 4 (rigid in flexion or extension). The MTS measured spasticity by moving the ankle slowly to record the passive range of motion (R2) and then rapidly to detect the catch angle (R1); the difference (R2–R1) reflected the dynamic component of spasticity. The TUG test was used to assess functional mobility. Participants stood up from a chair, walked three meters, turned around, walked back, and sat down. The total time was recorded, with shorter times indicating better mobility.

To ensure consistent data collection, ultrasonographic measurements (MFL, PA, strain elastography) and clinical outcome assessments (MAS, MTS, PROM, TUG test, and Barthel Index) were conducted at the same predefined time points: baseline, and weeks 1, 4, 12, and 24 after the intervention.

### Data analysis

Continuous data are presented as mean ± standard deviation (SD), ordinal variables as medians and interquartile ranges, and categorical variables as percentages. An intention-to-treat analysis was applied, with all randomized participants analyzed according to their originally assigned groups. For participants lost to follow-up, missing data were handled using available case analysis without imputation. Normality of data distribution was evaluated using the Shapiro–Wilk test. The Mann–Whitney U test was employed to analyze asymmetric data, while the Friedman test was used for analyzing repeated non-parametric measurements. The generalized estimating equation (GEE) analysis was adopted to evaluate time, group, and group-time interactions. Pearson correlation analyses were performed to investigate the linear relationships between the ultrasonographic muscular outcomes (MFL, PA, and strain elastography) and clinical outcomes (including MAS, MTS, PROM, TUG test, and Barthel index) within each group, respectively. Prior to the analysis, the normality and linearity assumptions were assessed to confirm the suitability of using Pearson correlation. Statistical significance was set at *p* < 0.05, two-tailed. Analyses were performed using IBM SPSS Statistics version 22.

## Results

### Baseline characteristics

A total of 39 participants were analyzed, including 19 participants in the double-dose ESWT group and 20 participants in the control ESWT group. There were no significant differences between the groups in terms of age, sex, comorbidities, stroke type and duration, MAS scores, MTS, PROM, TUG test, Barthel Index, strain elastography, PA, or MFL prior to the intervention. Baseline demographics and ultrasonographic measurements were comparable between groups (Table 1).

### Summary of previously published spasticity and functional results

Since this study is a secondary analysis of a published trial, we briefly summarize the main results to provide context before presenting the current ultrasonographic findings. The initial analysis of this RCT, published in our previous paper, demonstrated that double-dose ESWT significantly improved spasticity scores and functional performance in patients with post-stroke ankle plantar flexor spasticity [26]. Specifically, the double-dose ESWT group showed significant improvements in the TUG test, Barthel Index, and strain elastography compared to the control ESWT group [26]. The present analysis focuses on changes in ultrasonographic muscle architecture (MFL, PA, and strain elastography) and their associations with clinical outcomes.

**Table 1:** Baseline characteristics of the study participants

Characteristic	Double-dose ESWT group (*n* = 19)	Control ESWT group (*n* = 20)	*p*-value^†^
Age (years), mean (SD)	60.3 (14.3)	64.4 (10.3)	0.359
Female (%)	31.3	38.9	0.407
Hypertension (%)	87.5	88.9	0.926
DM (%)	18.8	27.8	0.558
Stroke onset (months), mean (SD)	17.3 (36.5)	22.8 (23.4)	0.595
Ischemic stroke (%)	68.8	38.9	0.089
Concomitant therapies (%)			
Physiotherapy	100	100	-
Ankle-foot orthosis	36.8	20.0	0.301
Anti-spastic drugs use	31.6	40.0	0.584
Modified Ashworth Scale, median (IQR)	2.63 (1.09)	2.56 (0.92)	0.815
Modified Tardieu Scale^∮^, mean (SD)	16.44 (8.82)	12.50 (11.01)	0.262
PROM (degrees), mean (SD)	44.67 (9.57)	44.72 (9.31)	0.986
Timed Up & Go Test (sec), mean (SD)	44.24 (38.82)	39.04 (29.71)	0.621
Barthel index, mean (SD)	68.13 (26.20)	66.39 (22.67)	0.871
Strain elastography, mean (SD)	1.09 (0.34)	1.10 (0.50)	0.846
PA (degrees), mean (SD)	15.58 (4.78)	16.91 (4.15)	0.433
MFL (cm), mean (SD)	4.56 (0.53)	4.3 (0.45)	0.285

Data in Table 1 are derived from the *Comparing ESWT Doses for Post-Stroke Ankle Spasticity Treatment* trial [25]

*ESWT* extracorporeal shockwave therapy, *SD* standard deviation, *DM* diabetes mellitus, *PROM* passive range of motion, *sec* seconds, *PA* pennation angle, *MFL* muscle fascicle length, *cm* centimeters

† Between-group comparison: Mann-Whitney U test was used for statistical analysis

∮ Modified Tardieu Scale was presented with R2-R1 Angle (degrees) where R1 was the angle of catch seen at quick speed and R2 was the full range of motion at slow release of muscle

**Table 2:** Improvements of outcomes in both groups: within-group analysis

Outcome	Baseline	Week 1	Week 4	Week 12	Week 24	*p*-value^†^
*PA*						
Double-dose ESWT group	15.58 (4.78)	16.13 (3.81)	16.12 (3.83)	16.46 (4.31)	15.02 (3.89)	0.458
Control ESWT group	16.91 (4.15)	15.87 (2.88)	16.59 (2.6)	15.91 (3.43)	16.99 (1.95)	0.861
*MFL*						
Double-dose ESWT group	4.56 (0.53)	4.83 (0.69)	4.32 (0.81)	4.68 (0.62)	4.27 (0.36)	0.938
Control ESWT group	4.30 (0.45)	4.57 (0.59)	4.50 (0.76)	4.56 (0.77)	4.23 (0.54)	0.177

The data are presented as the mean (SD)

*PA* pennation angle, *ESWT* extracorporeal shockwave therapy, *MFL* muscle fascicle length, *SD* standard deviation

† Friedman test analysis was used for repeated measurements of non-parametric comparison

*p < 0.05, **p < 0.01

**Table 3:** Effect of shockwave on outcomes between groups and over time

Ultrasound parameters	*p*-value^†^		
	Group	Time	Group x time
PA	0.585	0.956	0.569
MFL	0.340	0.007**	0.772
Strain elastography	0.222	0.209	0.008**

Strain elastography data are derived from the *Comparing ESWT Doses for Post-Stroke Ankle Spasticity Treatment* trial [25]

The data are presented as the mean (SD)

*PA* pennation angle, *MFL* muscle fascicle length, *SD* standard deviation

† Generalised estimation equation analysis was used for between-time, between-group, and group-time interaction

*p < 0.05, **p < 0.01

**Table 4:** Correlation between ultrasonographic muscular outcomes and clinical outcomes in both groups

Clinical outcomes (*R*, *p* value)	Double-dose ESWT group			Control ESWT group		
	Elastography	PA	MFL	Elastography	PA	MFL
MAS	− 0.017, 0.870	0.232, 0.032*	− 0.210, 0.156	− 0.073, 0.488	0.003, 0.982	− 0.207, 0.145
MTS^†^	0.185, 0.072	− 0.080, 0.469	0.300, 0.040*	− 0.125, 0.234	− 0.01, 0.931	− 0.123, 0.392
PROM	0.164, 0.111	− 0.129, 0.240	0.423, 0.003**	− 0.094, 0.374	0.045, 0.704	0.063, 0.663
Timed Up and Go test	− 0.149, 0.148	0.077, 0.481	− 0.644, < 0.001**	− 0.004, 0.056	− 0.105, 0.374	− 0.245, 0.084
Barthel index	0.017, 0.870	− 0.006, 0.956	0.063, 0.683	0.124, 0.237	0.171, 0.144	0.296, 0.053

Clinical outcomes and strain elastography data are derived from the *Comparing ESWT Doses for Post-Stroke Ankle Spasticity Treatment* trial [25]

*R* correlation coefficient, *ESWT* extracorporeal shockwave therapy, *PA* pennation angle, *MFL* muscle fascicle length, *MAS* Modified Ashworth Scale, *MTS* Modified Tardieu Scale, *PROM* passive range of motion

† Modified Tardieu Scale was presented with an R2-R1 angle (degree), where R1 was the angle of catch seen at quick speed and R2 was the full range of motion at slow release of muscle

*p < 0.05, **p < 0.01

### Changes in muscle architecture within each group

This section presents the within-group comparisons of PA and MFL across the 24-week follow-up period after ESWT (Table 2). In the double-dose ESWT group, the mean PA increased slightly from 15.58 degrees (SD = 4.78) at baseline to 15.02 degrees (SD = 3.89) at week 24. In the control ESWT group, the mean PA changed from 16.91 degrees (SD = 4.15) to 16.99 degrees (SD = 1.95) over the same period. The Friedman test showed no significant within-group changes in PA for either group (*p* = 0.458 for the double-dose ESWT group and *p* = 0.861 for the control ESWT group).

For MFL, the double-dose ESWT group showed an increase from 4.56 cm (SD = 0.53) at baseline to 4.83 cm (SD = 0.69) at week 1, followed by a decrease to 4.27 cm (SD = 0.36) at week 24. Similarly, the control ESWT group showed an increase from 4.30 cm (SD = 0.45) to 4.57 cm (SD = 0.59) at week 1, then decreased to 4.23 cm (SD = 0.54) by week 24. The within-group analysis revealed no statistically significant changes in MFL over time in either group (*p* = 0.938 for the double-dose ESWT group and *p* = 0.177 for the control ESWT group).

In summary, no significant longitudinal changes in PA or MFL were observed from baseline in either the double-dose or control ESWT groups. Although these within-group changes were not statistically significant, the early increases in MFL observed at week 1 in both groups may indicate a short-term adaptation of muscle architecture to ESWT, potentially associated with transient reductions in muscle stiffness or improvements in muscle extensibility. The absence of sustained or significant changes over time suggests that these effects may be modest or vary among individuals.

### Between-group comparisons of muscle architecture changes over time

This section compares the effects of different ESWT doses on muscle architecture changes between the double-dose and control ESWT groups at each follow-up time point (Table 3) by GEE analysis. The analysis revealed no significant group effect on PA (*p* = 0.585), MFL (*p* = 0.340), and strain elastography (*p* = 0.222). There was also no significant time effect on PA (*p* = 0.956) or strain elastography (*p* = 0.209). However, a significant time effect was found for MFL (*p* = 0.007), indicating modest increases in MFL across participants regardless of group allocation. Nevertheless, the magnitude of change was small, suggesting that the observed difference, while statistically significant, may have limited clinical impact. No significant interaction between group and time was found for PA (*p* = 0.569) or MFL (*p* = 0.772), indicating similar trends over time in both groups. In contrast, a significant group-time interaction was observed for strain elastography (*p* = 0.008), suggesting that changes in muscle stiffness differed between the double-dose and control ESWT groups during the study period.

### Correlations between ultrasonographic muscle outcomes and clinical measures

This section explores the associations between muscle architectural properties and clinical outcomes (MAS, MTS, PROM, TUG test, and Barthel Index) within each group, with a particular focus on differences in correlation patterns between the double-dose and control ESWT groups (Table 4) by Pearson correlation analyses. In the double-dose ESWT group, MFL demonstrated significant positive correlations with MTS (*R* = 0.300, *p* = 0.040) and PROM (*R* = 0.423, *p* = 0.003), as well as a negative correlation with the TUG test (*R* = − 0.644, *p* < 0.001), indicating that longer fascicle length was associated with improved spasticity measures, greater ankle range of motion, and faster TUG times. Additionally, PA exhibited a significant correlation with the MAS (*R* = 0.232, *p* = 0.032), suggesting that increased PA was linked to greater spasticity. In contrast, in the control ESWT group, no significant correlations were found between ultrasonographic outcomes and clinical measures. For example, MFL did not correlate significantly with MAS (*R* = − 0.207, *p* = 0.145), MTS (*R* = − 0.123, *p* = 0.392), PROM (*R* = 0.063, *p* = 0.663), TUG test (*R* = − 0.245, *p* = 0.084), or Barthel Index (*R* = 0.296, *p* = 0.053). Similarly, PA and elastography showed no statistically significant associations with any clinical outcomes in the control ESWT group. These findings suggest that associations between muscle architectural characteristics and clinical outcomes appeared more prominently in the double-dose ESWT group. Although these correlations do not imply a causal relationship, they may indicate potential dose-related patterns.

## Discussion

This study explored how different doses of focused ESWT influence ultrasonographic muscle properties in patients with post-stroke ankle spasticity. As no statistically significant between-group differences were observed for PA, MFL, or strain elastography, the following interpretations should be regarded as exploratory and interpreted with caution. Nevertheless, the overall improvement in MFL over time suggests a potential benefit of ESWT in promoting muscle architectural recovery. Moreover, the observed group–time interactions for strain elastography imply that higher ESWT doses might help maintain muscle elasticity over the longer term. Importantly, significant correlations found in the double-dose ESWT group between MFL and key functional measures, including the MTS, PROM, and TUG test, suggest that muscle architecture improvements could translate into clinically meaningful gains such as reduced spasticity, enhanced ankle mobility, and improved walking performance. Likewise, the association between PA and MAS supports the value of ultrasonographic parameters as complementary tools to monitor treatment responses in stroke rehabilitation.

In this study, the baseline values of MFL and PA of the spastic medial gastrocnemius were comparable with those reported in previous studies (MFL: 32–59 mm; PA: 14–37°) [3, 29, 30]. Since our study did not include healthy controls, we lack information on whether the values observed differed among stroke survivors, healthy individuals, or healthy limbs. However, there is ongoing debate regarding the architectural changes of spastic muscles as detected by ultrasonography in patients with chronic stroke. Some studies reported shorter MFL in spastic muscles compared to healthy individuals [3, 31], while others found no significant differences between paretic, non-paretic, and healthy limbs [28]. Similarly, for PA, some studies suggested smaller angles in hemiplegic legs compared with non-hemiplegic or healthy legs [30–32], but other studies showed no significant differences [5, 33]. A systematic review also reported this inconsistency, indicating that variations in methodology and participant characteristics limited the ability to draw definitive conclusions regarding MFL and PA changes in stroke populations [34].

Despite the limited ultrasonographic evidence, additional literature on spasticity-related neurological diseases supports the hypothesis of shortened MFL in spastic muscles. Friden et al. conducted experiments on single muscle fiber segments obtained from patients with cerebral palsy undergoing surgical correction for contracture and found shorter resting sarcomere lengths and a two-fold increase in elastic modulus [35]. Additionally, decreased fascicular lengths in the soleus and gastrocnemius muscles have been reported in children with cerebral palsy, as measured by ultrasonography [36]. Further studies are required to validate this hypothesis with a more homogeneous study group and consistent methodology.

Our study found no significant changes in MFL or PA in either the double-dose ESWT group or the control ESWT group. Despite the abundant literature focusing on the clinical and biomechanical outcomes of ESWT for treating spasticity, only two studies, to our knowledge, have evaluated its effects on muscular architecture. An RCT employing a single session of ESWT targeting the medial gastrocnemius in patients with chronic stroke demonstrated improvements in MAS scores and significant changes in ultrasonographic measurements, including shorter MFL and smaller PA at weeks 1 and 4 post-intervention [21]. In contrast, our study employed a substantially higher dose of intervention (2000/4000 shots, totaling four sessions) but showed non-significant results. The discrepancy may have originated from differences in participant characteristics, such as older age and longer post-stroke duration in our study, or from variations in the ultrasonographic scanning reference points. However, these remain speculative explanations that require further investigation and should not be considered definitive causes. The results of another single-group study were more consistent with our study, showing no significant changes in MFL and PA in patients with chronic stroke after a single session of ESWT with 2000 shots on the affected ankle plantar flexor muscles [10]. Notably, that study only evaluated outcomes immediately and 30 min following the intervention. The absence of long-term evaluation may obscure the potential long-term benefits of muscle tone reduction observed at 4 to 12 weeks in previous studies [37, 38].

The mechanisms through which ESWT may alleviate spasticity are likely multifactorial and remain incompletely understood. Neurophysiological evaluations have indicated that ESWT does not significantly affect alpha motor neuron excitability, which leads to the hypothesis that its clinical improvements may instead arise from changes in connective tissue stiffness and muscle architecture than from direct neural effects [37, 39]. Previous research applying ESWT in patients with PSS has yielded mixed results, potentially due to variations in shockwave generation methods, energy levels, application frequency, treatment sites, and illness duration [8, 40]. Spasticity involves both stretch reflex and muscle stiffness components. The stretch reflex heightens within 1 to 3 months post-stroke and then declines, whereas passive muscle stiffness progressively increases [41]. The duration of illness is crucial for ESWT efficacy; prolonged illness leads to persistent and worsening tissue stiffness, impacting treatment outcomes [42]. Earlier studies have reported mean times since stroke of 22.0 ± 8.2 months (Bae et al.) [42], 9.5 ± 6.8 months (Yoo et al.) [43], and 12.9 ± 8.9 months (Lee et al.) [21], with varying treatment effects lasting up to four weeks. In our study, the mean duration since stroke was 17.3 months for the double-dose ESWT group and 22.8 months for the control ESWT group. These differences in patient characteristics, stroke chronicity, and ESWT protocols may have contributed to the variability of results. Our findings highlight the need for further research to determine optimal ESWT protocols and investigate long-term effects on muscle architecture and spasticity.

In this study, we found an association between changes in ultrasonographic muscle structures and clinical outcomes. Previous research has established a link between spasticity scores and the muscle architecture of the spastic medial gastrocnemius muscle [5, 10]. For instance, Rastgoo et al. demonstrated a positive correlation between improvements in ankle PROM and fascicle length in patients with stroke who received 2000 shots of ESWT on the affected side in one session [10]. Conversely, Lee et al. found no significant relationship between changes in clinical measures and MFL [21]. Our study revealed that MFL was significantly associated with several clinical measures, including the MTS, PROM, and the TUG test in the double-dose ESWT group. The negative correlation between MFL of the paretic medial gastrocnemius and TUG performance suggests that shorter fascicle length may be associated with reduced ankle mobility and impaired gait efficiency. Specifically, reduced MFL may reflect structural adaptations in the muscle, such as increased stiffness or fibrotic changes, which can limit its ability to elongate effectively during gait. We hypothesized that limited fascicle extensibility may restrict dorsiflexion during stance and compromise plantarflexion force generation during the terminal stance and push-off phases, resulting in reduced propulsion, slower gait velocity, and decreased dynamic balance. These biomechanical impairments collectively contribute to a slower TUG performance and increased fall risk. Therefore, sonographic assessment of MFL may provide useful clinical information about functional mobility in patients with post-stroke ankle spasticity. The GEE analysis also revealed a significant effect of time on MFL, indicating overall improvements in both groups. This could be due to the fact that both the double-dose ESWT group and the control ESWT group received 2000 shots on the gastrocnemius, leading to similar fascicle lengthening effects. However, the double-dose ESWT group showed better functional improvements, such as in the TUG test, indicating higher effectiveness of the ESWT dose. The increase in MFL observed over time suggests that repeated shockwave therapy may promote muscle lengthening, potentially enhancing the range of motion and improving functional mobility and balance abilities. The correlation between MFL and clinical measures such as MTS, PROM, and the TUG test indicates that longer muscle fascicles are associated with better functional outcomes. This suggests that ESWT may induce structural changes in muscles that contribute to functional improvements.

Regarding PA, the relationship between PA and post-stroke muscle property changes remains inconclusive [5, 30–33]. Ramsay et al. reported a significant decrease in the paretic medial gastrocnemius PA compared to both nonparetic and healthy limbs [28]. They explained this by the transmitted muscular force along the axis of the fascicle associated with PA. Smaller PA may indicate muscle atrophy in the paretic limb [28]. Picelli et al. investigated the relationship between clinical and ultrasonographic indicators in adult patients with stroke exhibiting spastic equinus and found that the MAS score was inversely related to PA [5]. This contrasts with our findings, where larger PAs were associated with increased muscle tone. Similarly, Yang et al. demonstrated a positive correlation between MAS and PA, explaining that spasticity causes muscle contractions and increased vertical torque to the fascicle plane as well as PA [4]. Additionally, the increase in PA may reflect compensatory mechanisms in response to spasticity. One ultrasound study revealed greater stiffness of the tibialis anterior muscle as PA increased [44]. Another possible reason for these inconsistent results is that the observed PA differences in our study were smaller than 5°, which is functionally negligible due to the minimal change in muscle force transmission at such small angles [21]. Similarly, Maisetti et al. reported that PA variations within 10° have little effect on muscle stiffness measurements of the gastrocnemius [45]. Further studies are needed to better clarify the relationship between PA and spasticity after stroke.

Although the intervention period in this study was relatively short at two weeks, the follow-up duration was extended to 24 weeks. This approach was based on treatment protocols commonly used in prior ESWT studies, which typically ranged from one to four weeks with a total of one to four treatment sessions [9, 46, 47]. We adopted a regimen of four sessions over two weeks, consistent with previous investigations [46]. However, since most earlier studies did not evaluate outcomes beyond 12 weeks [9], we extended the follow-up period to 24 weeks to better assess the potential durability and sustainability of treatment effects over the longer term.

A key strength of this study is the detailed ultrasonographic evaluation of muscle architectural changes, which provides objective, quantifiable evidence of muscle adaptation following ESWT in patients with post-stroke ankle plantar flexor spasticity. By focusing on MFL, PA, and strain elastography, this study presents sonographic findings that complement traditional clinical assessments and may reveal subtle structural improvements not captured by spasticity scales alone. While the double-blind, randomized controlled design and dose-response effect were established in our previous work, the present analysis further extends our understanding by directly linking muscle architecture changes with functional outcomes, supporting the clinical relevance of ultrasonographic monitoring in spasticity management.

### Limitations

Our study has several limitations. First, we did not include healthy controls, which limits our ability to compare ultrasonographic findings between stroke survivors and healthy individuals. Second, the heterogeneity of our study population, including older patients with varying durations post-stroke, may have contributed to inconsistent results. Moreover, since all participants were post-stroke patients within a defined recovery phase, the generalizability of our findings is limited to this specific population and should not be extended to individuals with spasticity of other etiologies or those in different stages of recovery. Third, our study employed a higher ESWT dose and more treatment sessions compared to previous studies. While we followed participants for up to 24 weeks, other studies typically have a follow-up period of only 4 weeks, making it challenging to compare the effectiveness of different ESWT doses and their effects over time. Fourth, there was approximately a 15% loss to follow-up in each group at the 12- and 24-week assessments, which may have introduced potential bias in the long-term outcome comparisons. Since we used available case analysis without imputation, the results should be interpreted with caution, especially regarding the long-term effects. Additionally, the use of a single assessor for all ultrasound measurements could introduce potential measurement bias, even though intra- and inter-rater reliability was assessed. Lastly, this study did not include neurophysiological assessments, such as H-reflex measurements, to evaluate changes in alpha motor neuron excitability. Therefore, no direct evidence regarding the neural mechanisms of ESWT can be drawn from our results. Further research is warranted to standardize protocols, include healthy controls, incorporate neurophysiological assessments, and conduct long-term evaluations to better understand ESWT’s effects on ultrasonographic muscle properties and spasticity.

## Conclusions

No significant differences in PA or MFL were found between the double-dose and control ESWT groups; however, MFL significantly improved over time in all participants. Notably, in the double-dose ESWT group, improvements in ultrasonographic muscle properties, particularly MFL, were significantly correlated with better clinical outcomes, including faster TUG performance, MTS and greater ankle PROM, suggesting enhanced mobility, spasticity control and ankle flexibility. Although definitive dose-response effects between groups were not statistically demonstrated, these findings indicate that focused ESWT may not only induce structural muscle changes but also contribute to meaningful functional recovery in patients with ankle plantar flexor spasticity after stroke. Ultrasonographic evaluation of muscle architecture may offer a useful adjunctive tool for monitoring treatment effects. Future research should investigate the long-term outcomes of various ESWT dosing strategies, explore the underlying physiological mechanisms, and confirm these results through large-scale, multicenter trials.

## Supplementary Information

Below is the link to the electronic supplementary material.

Supplementary Material 1.

Supplementary Material 2.

## Data Availability

No datasets were generated or analysed during the current study.
