# Supplementary figures and images for: Dose–response effectiveness of focused shockwave therapy on ultrasonographic muscular properties in patients with stroke exhibiting ankle spasticity
Source: J Neuroeng Rehabil. 2025 Aug 21;22:184. doi: 10.1186/s12984-025-01724-7 (PMC12372182; doi:10.1186/s12984-025-01724-7)

**S-Figure 1.**


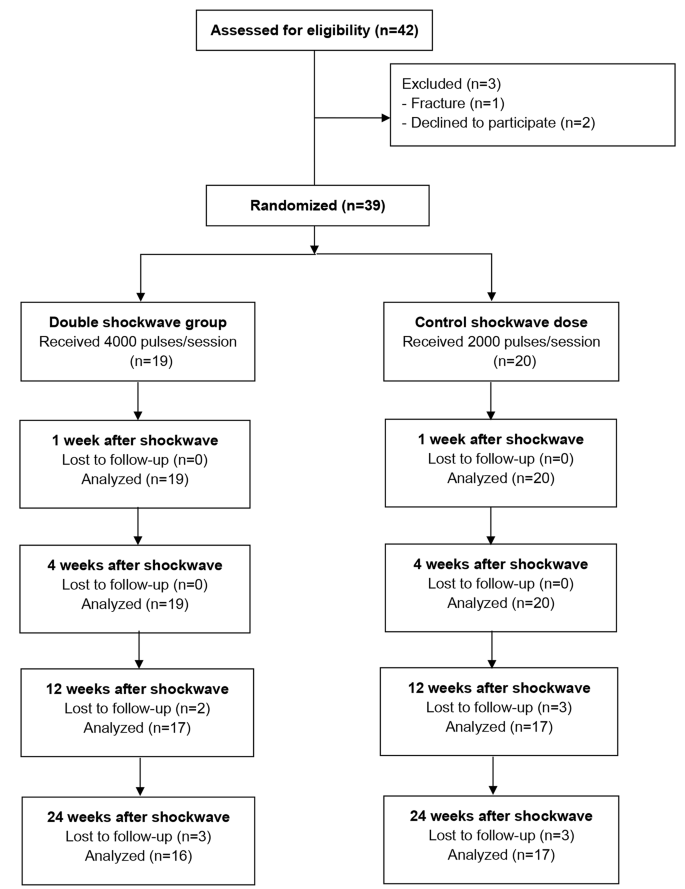

Supplement: Supplementary file 1 — Supplementary Material 1. [file 12984_2025_1724_MOESM1_ESM.docx]
